# The Timing of Change Detection and Change Perception in Complex Acoustic Scenes

**DOI:** 10.3389/fpsyg.2012.00396

**Published:** 2012-10-12

**Authors:** Zahrah Jaunmahomed, Maria Chait

**Affiliations:** ^1^UCL Ear InstituteLondon, UK

**Keywords:** time perception, change detection, audio-visual temporal order judgment, auditory scene analysis

## Abstract

We investigated how listeners perceive the temporal relationship of a light flash and a complex acoustic signal. The stimulus mimics ubiquitous events in busy scenes which are manifested as a change in the pattern of on-going fluctuation. Detecting pattern emergence inherently requires integration over time; resulting in such events being detected later than when they occurred. How does delayed detection time affect the perception of such events relative to other events in the scene? To model these situations, we use rapid sequences of tone pips with a time-frequency pattern that changes from random to regular (“REG-RAND”) or vice versa (“RAND-REG”). REG-RAND transitions are detected rapidly, but RAND-REG take longer to detect (∼880 ms post nominal transition). Using a Temporal Order Judgment task, we instructed subjects to indicate whether the flash appeared before or after the acoustic transition. The point of subjective simultaneity between the flash and RAND-REG does not occur at the point of detection (∼880 ms post nominal transition) but ∼470 ms closer to the nominal acoustic transition. In a second experiment we halved the tone pip duration. The resulting pattern of performance was qualitatively similar to that in Experiment 1, but scaled by half. Our results indicates that the brain possesses mechanisms that survey the proximal history of an on-going stimulus and automatically adjust perception so as to compensate for prolonged detection time, thus producing more accurate representations of scene dynamics. However, this readjustment is not complete.

## Introduction

In order to successfully interact with our surroundings, we must be able to rapidly and accurately detect new events or changes in the scene. Hearing plays a key role in this process – It samples a broader sphere of events than the other senses, and also elicits the fastest behavioral response time (RT; Luce, 1986). Indeed, it is widely hypothesized that the auditory system possesses specialized, automatic, highly tuned mechanisms for change detection (e.g., Ulanovsky et al., 2003; Chait et al., 2007; Näätänen et al., 2007).

Many natural acoustic environments are “busy” – containing a cacophony of multiple, simultaneous, sources. The detection of new events in such scenes cannot be accomplished by simply detecting sound onset (transition from silence to sound) but rather involves the identification of a change in the on-going sound pattern (Chait et al., 2008). In some cases the new event consists of an absolute change in some feature (e.g., a different frequency, increase in loudness, etc.) and can therefore be detected rapidly. However many other events, equally pervasive in natural acoustic scenes, do not involve an outright change in power but are rather characterized by a change in the *statistics* of fluctuation (the arrival of a bus along a curb, from which one was about to step, produces merely a change in the pattern of the on-going brouhaha of a busy city). The detection of change in fluctuation pattern involves accumulating information over a certain duration, and thus necessarily takes more time. This, in turn, results in a potential “scene analysis” failure – namely, a class of acoustic events that are detected much later than when they actually occurred.

The issue of detection time is profoundly important. Organisms critically rely on their perception of the timing of events in order to operate effectively and efficiently in the environment – avoid predators and capture pray. An accurate model of the absolute- (“*when?*”) and relative- (“*which came first?*”) timing of events provides key information about scene dynamics that is vital for making sense of the world around us – e.g., determining cause and effect or deciding whether different sensory events should be bound to a unified multi-sensory representation. How, then, does the brain deal with rather common situations, where there exists a significant mismatch between when an event has been detected and when it actually occurred? Is the brain able to “realize” that its detection time is inaccurate relative to occurrence time and correct perception accordingly?

**Figure 1:**
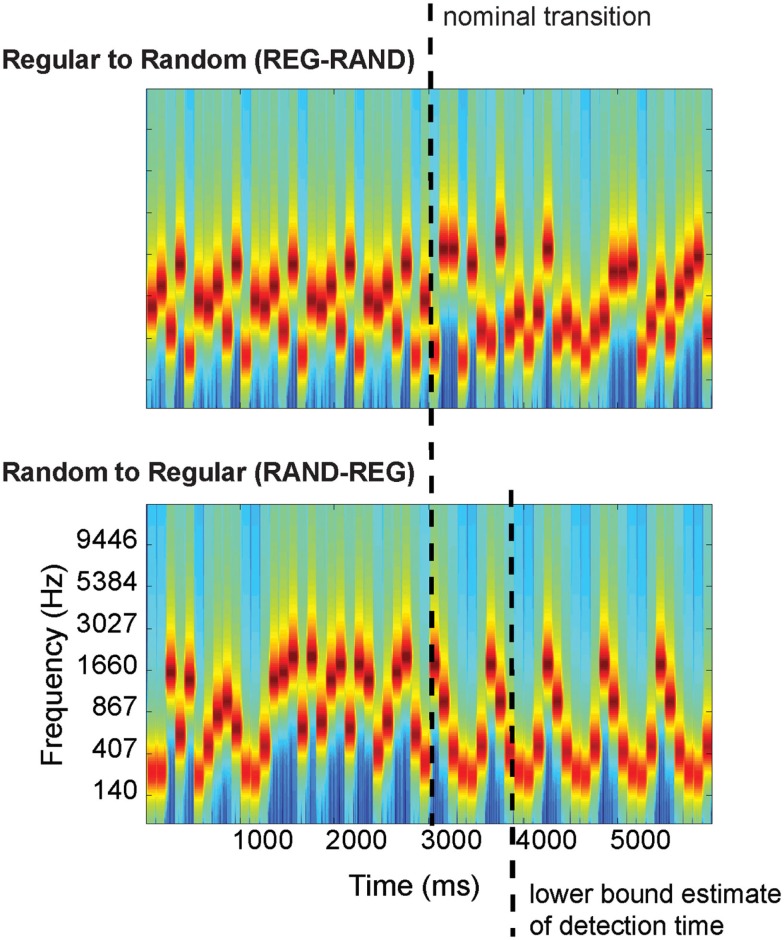
**Examples of the auditory stimuli**. Top: transition between a regular and random frequency pattern (REG-RAND). Bottom: transition between a random to regular frequency pattern (RAND-REG). The nominal transition, and lower bound estimate of detection time (for RAND-REG; derived in the DETECT block) are indicated with dashed lines. The plots represent “auditory” spectrograms, generated with a filterbank of 1/ERB wide channels (Equivalent Rectangular Bandwidth; Moore and Glasberg, 1983) equally spaced on a scale of ERB-rate. Channels are smoothed to obtain a temporal resolution similar to the Equivalent Rectangular Duration (Plack and Moore, 1990).

We have recently proposed a paradigm for measuring the timing of change perception (*when the change is perceived to have occurred*) relative to the timing of change detection (*when the change was first detected*; Patel and Chait, 2011). To model complex acoustic events with different detection times, we use tone pip sequences that contain transitions between random and regular frequency patterns (Figure 1). Transitions from a regularly alternating to a random tone sequence (REG-RAND) are immediately detectable as the first tone to violate the established regularity pattern is sufficient to signal the transition. In contrast, the opposite transition – from a random to a regular pattern (RAND-REG) – requires more time to detect because listeners must wait long enough (>1 regularity cycle) to discover the regular pattern. This duration depends on the statistical properties of the signal before/after the transition and also on a decision criterion (namely, how much evidence one considers as sufficient in order to decide that the pattern is repeating).

The sequences we use are too rapid for conscious calculation (tone pip duration is 100 ms in Experiment 1, and 50 ms in Experiment 2, below) such that listeners are unable to detect the change by explicitly scanning/memorizing the unfolding sound pattern and searching for regularities (see also, Garner, 1951; Warren and Byrnes, 1975). Instead, the percept is that of the regular pattern automatically popping out of the sequence and “grabbing” attention (see Demo Sounds in Supplementary Material), suggesting that detection of regularity emergence, and the criterion-based decision process discussed above, occur at a pre-attentive stage of processing.

Indeed MEG brain imaging experiments with such stimuli (Chait et al., 2008) demonstrate that the auditory cortex detects the emergence of regular patterns automatically, and rapidly, even in the absence of directed attention (when listeners are actively engaged in an unrelated task). The point of detection, measured as the first brain response to the transition, occurs roughly a cycle and a half after the nominal transition time (a similar estimate is also obtained by measuring behavioral detection time; see below). For complex patterns this means that the time at which change is first detected by the brain is several hundreds of milliseconds *later* than when the change actually occurred.

However, once the regularity pattern has been discovered, and if the brain maintains some form of memory of the just-heard sequence, it might be able to reverse-scan the sequence and determine the timing of the nominal transition. This is the question at the basis of the present work – Do listeners perceive the change to have occurred at the point of detection, or do they re-adjust the perceptual estimate of the change time backward in time toward the nominal transition? To measure whether such readjustment indeed occurs, we employ a manipulation of a similar flavor to the classic Libet clock experiments (Libet et al., 1983): we use a light flash (which occurs at a random point in time around the transition in the REG-RAND and RAND-REG signals) as a temporal marker and instruct listeners to determine the timing of the flash relative to the acoustic transition. Specifically, we are interested in their performance on the RAND-REG condition, with REG-RAND, where no temporal adjustment is necessary, serving as a control. The RAND-REG and REG-RAND stimuli are matched in terms of their spectral content and overall temporal structure with the only difference being the “temporal fuzziness” of the transition.

In a previous experiment using a regularity cycle of 4 tones (Patel and Chait, 2011), we demonstrated that listeners indeed do not perceive the transition to have occurred at the time at which it was detected but rather, the perceived change onset time is backward readjusted (by about 300 ms) closer to the nominal transition time. The purpose of the present work is to re-visit these effects with a different (longer) repeating pattern and across two regularity conditions characterized by different detection times, in order to probe the temporal properties of the mechanisms via which the brain compensates for delayed detection time.

## Experiment 1

### Methods

#### Participants

Ten paid subjects (mean age 22.6, five males) participated in the experiment. One subject’s data were excluded from the analysis due to inability to perform the task (no response for over 50% of the trials). All participants reported normal hearing, and no history of neurological disorders. The experimental procedures were approved by the UCL ethics committee and written informed consent was obtained from each participant.

#### Stimuli

The auditory stimuli were sequences of 100 ms tone pips which were presented in two patterns (Figure 1): REG-RAND (“regular to random”) and RAND-REG (“random to regular”). Tone frequencies were drawn from a set of 20 values equally spaced (12%) on a logarithmic scale between 222 and 2000 Hz. The amplitude of each pip was shaped by initial and final 5 ms raised-cosine ramps. The REG-RAND stimulus consisted of a repeating sequence of six different frequencies (randomly drawn from the above set) immediately followed by a sequence of pips with random frequencies. The RAND-REG stimulus consisted of the reverse pattern – a random sequence of tone pips, followed by a sequence of six regularly repeating tone pips. The initial sequence (whether “regular” or “random”) consisted of between 30 and 45 tone pips (3000–4500 ms), making the time of the occurrence of a transition unpredictable. The duration of the post-transition sequence was also randomized between 24 and 34 tone pips (2400–3400 ms). In addition to these stimuli, the stimulus set also included a number (13%) of “sham trials,” with duration of 5400–7900 ms, where the stimulus contained no transition (e.g., either “constant” or “random” for the entire duration of the stimulus). The purpose of the “sham trials” was to ensure subjects were attentive to the stimuli and cautious with their responses.

The stimuli were created on-line at a sampling rate of 44.1 kHz, delivered to the subjects’ ears with Sennheiser HD555 headphones (Sennheiser, Germany) and presented at a comfortable listening level (self-adjusted by each listener). The inter-stimulus interval (ISI) was 2000 ms.

The presentation of the auditory and visual stimuli was controlled with the Cogent software^1^. Subjects were tested in a darkened, acoustically shielded room (IAC triple walled sound attenuating booth). They were seated about 70 cm away from a black computer screen (LCD, 60 Hz refresh rate) and asked to fixate at a cross (diameter 1 cm) at its center. The experiment consisted of two blocks. In the DETECT (20 min) block, 50 REG-RAND, 50 RAND-REG, and 16 sham trails were presented in random order. Subjects were asked to fixate at a white cross in the center of the computer screen while listening to the stimuli and instructed to respond, by pressing a keyboard button, as soon as they detected the stimulus transitions. They were asked to withhold a response during “sham trials.” The ORDER JUDGMENT (90 min) block, contained similar stimuli but at −1000 to +1500 ms around the transition in the auditory stimuli, the fixation cross changed to a white filled circle (diameter 2.5 cm) that was presented in the middle of the screen for a duration of 20 ms, resulting in the percept of a brief light flash. The light flash could coincide with any tone pip, from 10 pips before the transition and up to 14 pips after the transition. The visual display command was executed together with the onset of an auditory tone however due to the screen’s refresh rate the actual appearance of the visual stimulus had a variable latency of up to 20 ms. The flash was perceived as a single, clear, event (e.g., not affected by illusions such as those reported in Shams et al., 2002).

In the ORDER JUDGMENT block, subjects were instructed to perform a temporal order judgment (TOJ) task (single interval two alternative forced choice), whereby they had to determine whether the light flash appeared *before or after* the transition by pressing one of two keyboard buttons. Following each stimulus, a question mark, which lasted 1500 ms, appeared on the screen. Subjects were instructed to give their response during this interval and withhold responses during “sham trails.” The instructions given to subjects stressed the importance of responding rapidly and guessing when unsure about the temporal order.

It has been suggested that the TOJ task may be more susceptible to response bias or criterion shifts than a simultaneity judgment (SJ) task (Schneider and Bavelier, 2003; van Eijk et al., 2008). This is not a major concern for the present experiments because the REG-RAND stimuli served as “built in” controls for the RAND-REG stimuli. Any systematic response bias or bias in relative timing of auditory and visual events should cancel out, as long as this bias is the same for each auditory event (see also Discussion in Patel and Chait, 2011).

#### Procedure

A short practice session preceded the actual experiment. Data acquisition was divided into runs of about 10 min. Between runs, subjects were allowed a short rest.

#### Data analysis

**Figure 2:**
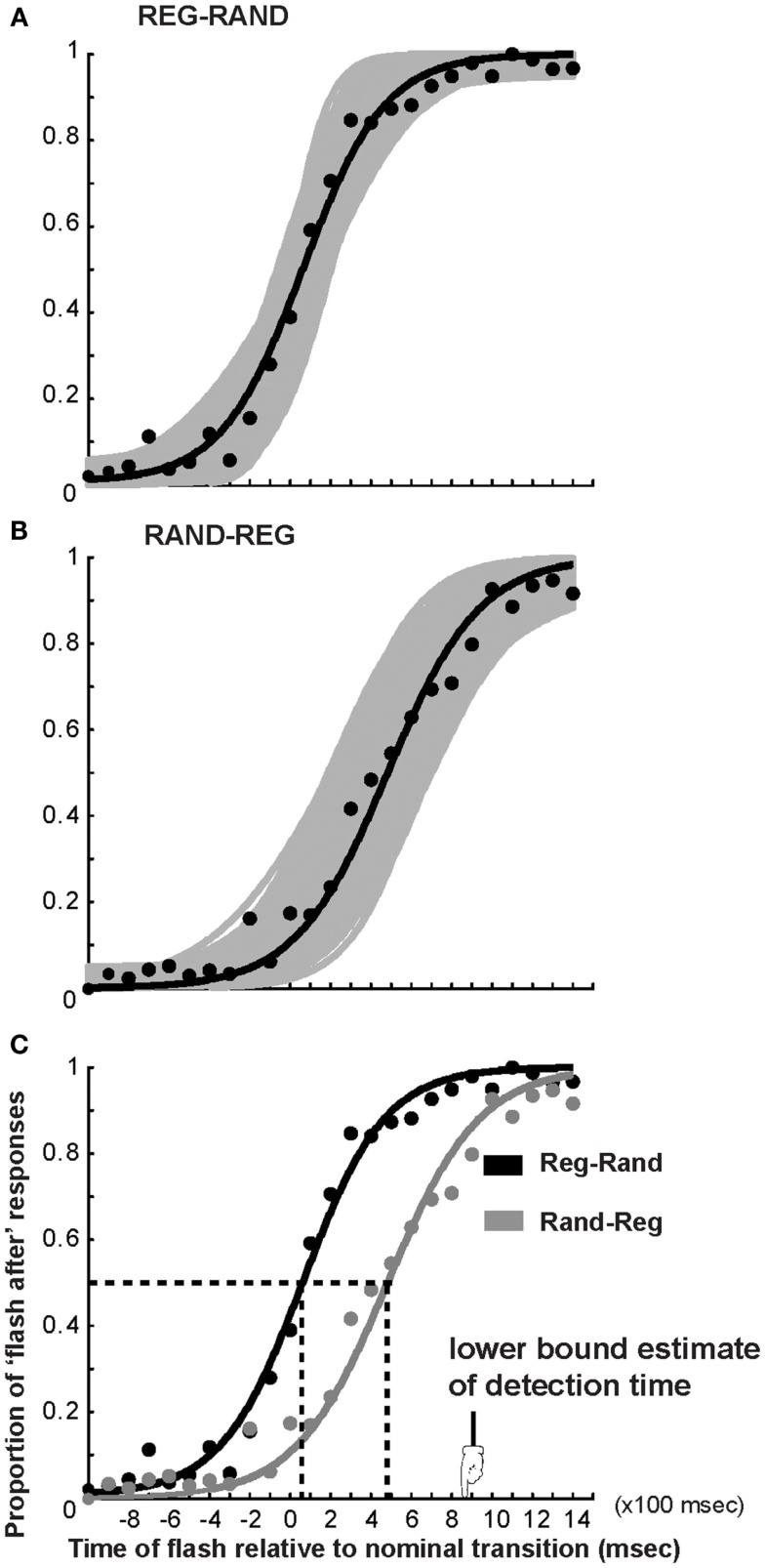
**Psychometric fits to the TOJ data in Experiment 1**. The *x*-axis represents the timing of the light flash with respect to the acoustic transition (measured in number of tone pips, multiply by 100 to obtain milliseconds). Negative numbers indicate flash before nominal transition. **(A)** Proportion of “light flash after” responses for the REG-RAND stimulus averaged over subjects (black circles) and cumulative Gaussian fit (black curve). The gray curves are fits computed over 1000 bootstrap resamplings; their scatter provides a measure of variability across subjects. **(B)** Same for the RAND-REG transition. **(C)** Comparison of REG-RAND and RAND-REG psychometric functions. The vertical dashed lines indicate the PSS.

**Figure 3:**
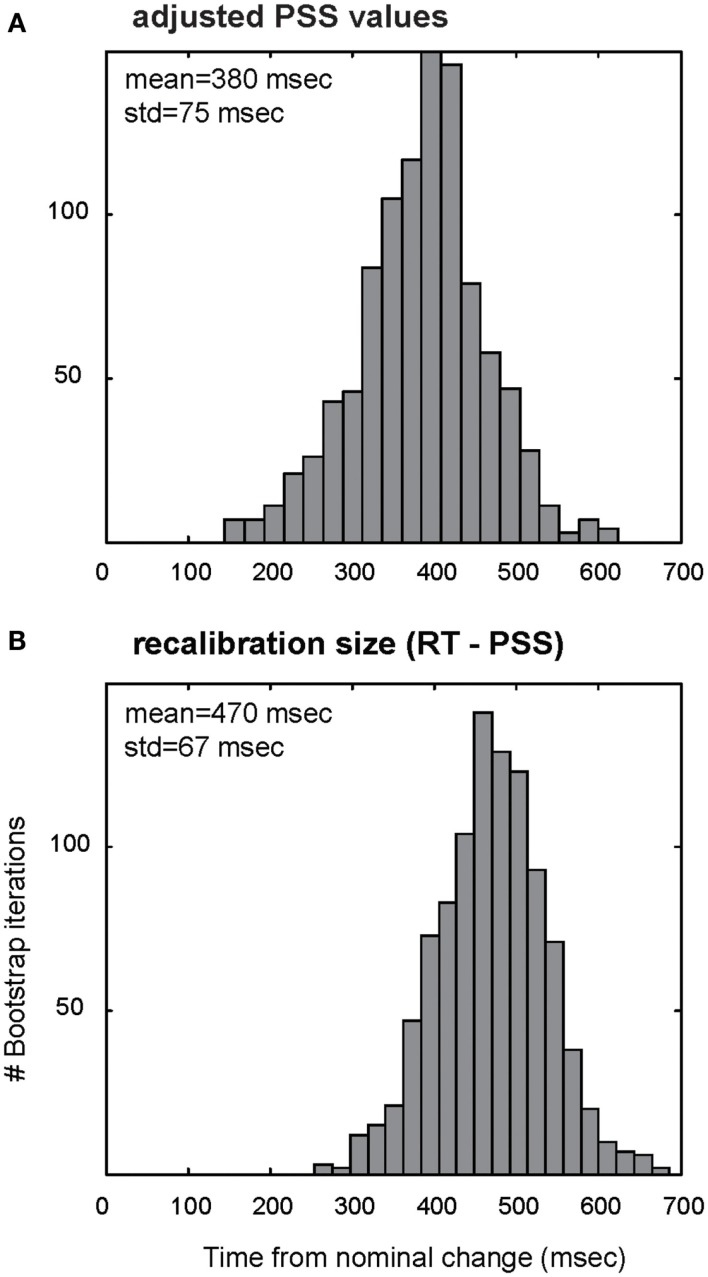
**Distributions of RAND-REG adjusted PSS values (A) and recalibration size (B) in Experiment 1 as computed with bootstrap resampling**. **(A)** Gray bars: histogram of average PSS values obtained with a “repeated measures” bootstrap procedure, by which we repeatedly select with replacement a set of 15 subjects, estimate each individual PSS (including correction of RAND-REG PSS by the PSS of REG-RAND), compute the average over the group, and increment the appropriate histogram bin. **(B)** Same for recalibration size (RT-PSS).

**Figure 4:**
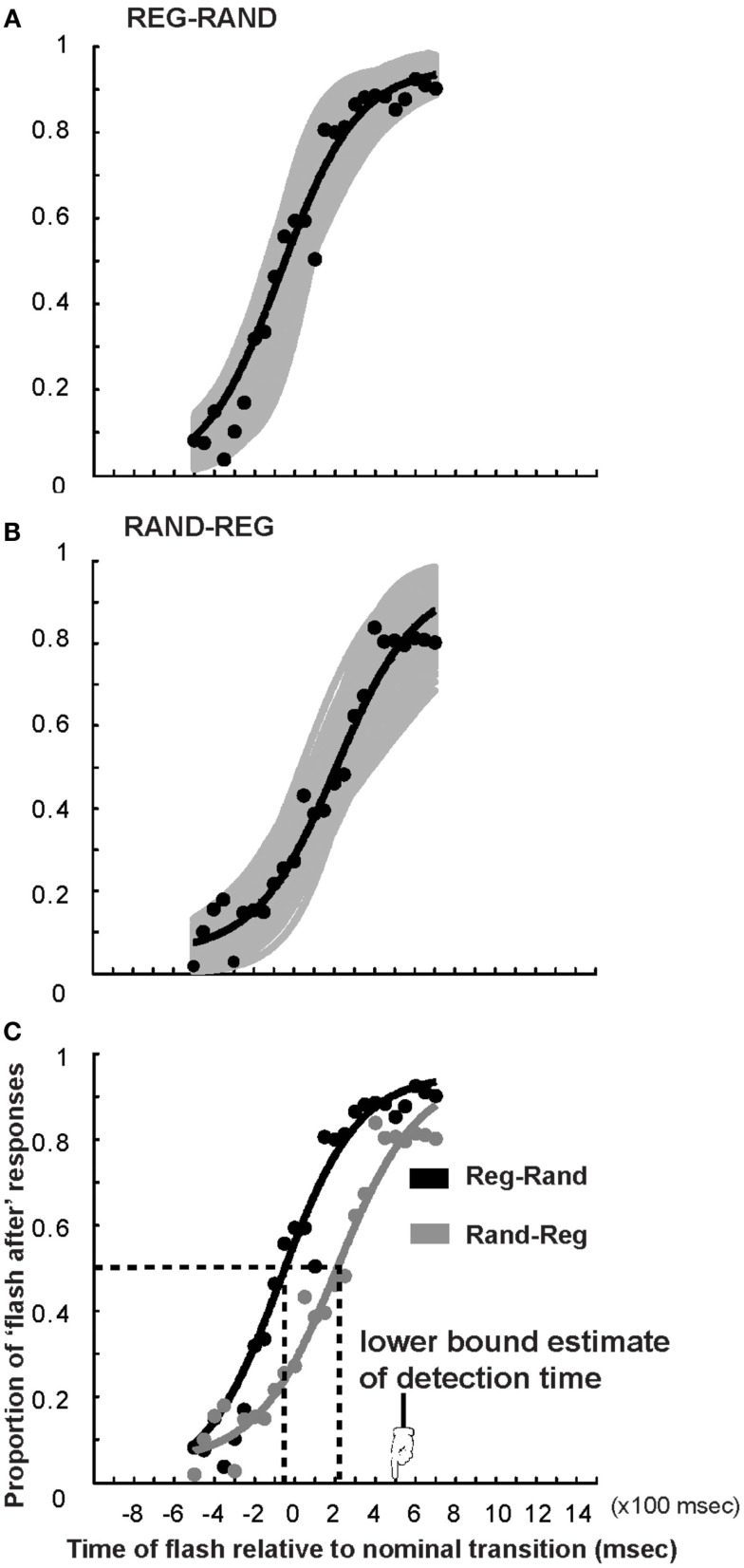
**Psychometric fits to the TOJ data in Experiment 2**. The *x*-axis represents the timing of the light flash with respect to the acoustic transition (measured in number of tone pips, multiply by 100 to obtain milliseconds). Negative numbers indicate flash before nominal transition. **(A)** Proportion of “light flash after” responses for the REG-RAND stimulus averaged over subjects (black circles) and cumulative Gaussian fit (black curve). The gray curves are fits computed over 1000 bootstrap resamplings; their scatter provides a measure of variability across subjects. **(B)** Same for the RAND-REG transition. **(C)** Comparison of REG-RAND and RAND-REG psychometric functions. The vertical dashed lines indicate the PSS.

**Figure 5:**
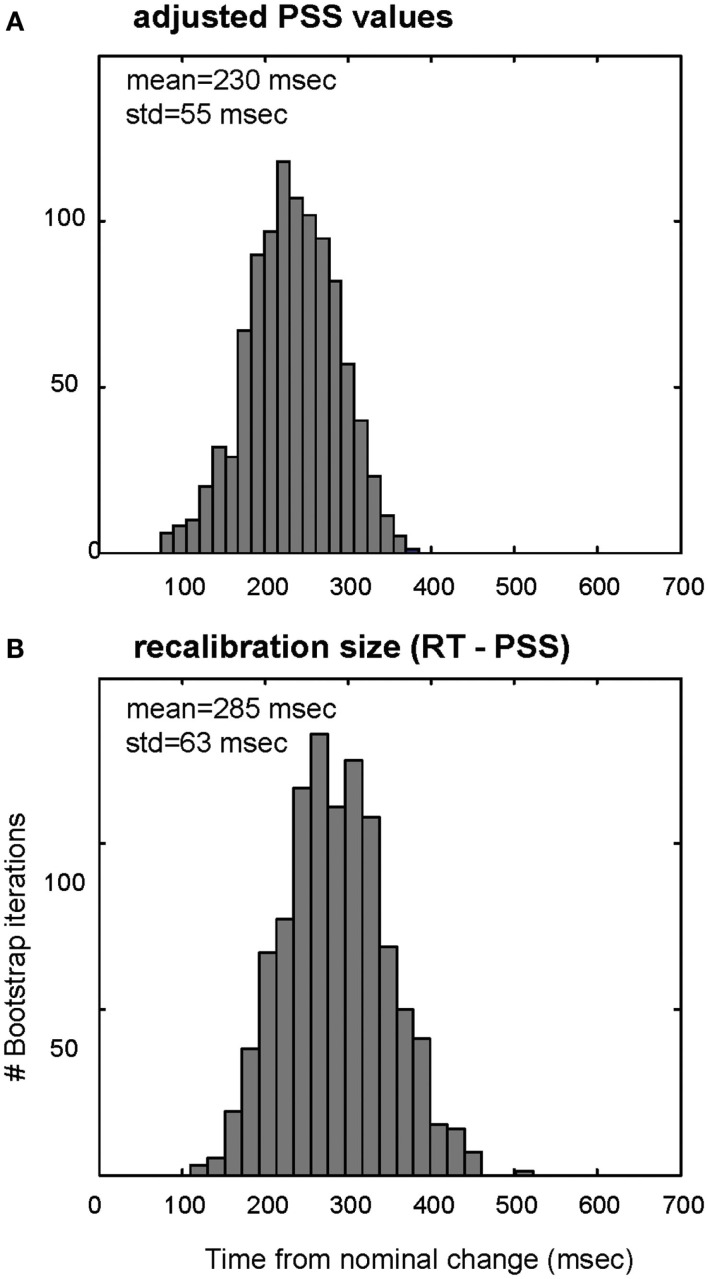
**Distributions of RAND-REG adjusted PSS values (A) and recalibration size (B) in Experiment 2 as computed with bootstrap resampling**. **(A)** Gray bars: histogram of average PSS values obtained with a “repeated measures” bootstrap procedure, by which we repeatedly select with replacement a set of 15 subjects, estimate each individual PSS (including correction of RAND-REG PSS by the PSS of REG-RAND), compute the average over the group, and increment the appropriate histogram bin. **(B)** Same for recalibration size (RT-PSS).

In the DETECT block, RTs were measured by calculating the latency between the onset of the transition and the subject’s key press. Responses with latency >2 SDs from the individual subject’s mean were excluded. In the ORDER JUDGMENT block, Psychometric functions (cumulative Gaussian) were fitted using the *psignifit* toolbox version 2.5.6 for Matlab^2^ which implements the maximum-likelihood method described in Wichmann and Hill (2001). To robustly estimate the point of subjective simultaneity (PSS) and it’s variability across participants, a bootstrap procedure was employed with 1000 iterations (Efron and Tibshirani, 1993). The bootstrap procedure allows to non-parametrically assess the mean and it’s variance without assuming an underlying Gaussian distribution. We estimated the PSS using two methods: (1) Based on the mean performance across subjects (Figures 2 and 4): In brief, we iteratively select with replacement a subset of N subjects (*N* = the number of participants), compute the average performance, fit the logistic function, and estimate the PSS. (2) Based on a within subjects, repeated measures analysis (Figures 3 and 5): we iteratively select N subjects, compute the individual psychometric fits for each condition in each subject, derive the corrected PSS, and then average that value across N.

### Results

#### The timing of change detection

In the DETECT block, the average RT measured for REG-RAND transitions was 702 ms (SD = 171), and that for RAND-REG was 1581 ms (SD = 100).

As discussed above, REG-RAND transitions are expected to be rapidly detected – the arrival of the first tone pip that violates the preceding regular pattern is enough to indicate to the listener that a change has occurred. On this basis, we consider that the RT latency measured for REG-RAND stimuli reflects the time it takes for the change to reach awareness, the time taken to program and/or generate the motor response, and the subject’s general state of vigilance (as well as hardware latency). The RT latency measured for the opposite transition, RAND-REG, presumably reflects those same processes as well as the extra processing time specific to that transition. Since the processing specific to the RAND-REG transition might overlap with processing common to both transitions, we consider the average RT difference between REG-RAND and RAND-REG stimuli – 879 ms – to be a *lower bound* measure of the computation time required to detect the emergence of the regular pattern in RAND-REG stimuli.

The difference in RT between conditions corresponds to a duration of about 9 tones. The regular pattern itself has a period of 6 tones, implying that on average, listeners required just three additional tones to recognize the onset of regularity. Presumably, to detect the regular pattern listeners must keep the recent stimulus history in working memory, compare incoming tones to the stored pattern and make a decision after several consecutive matches. The present data suggest that the auditory system is able to store sequences at least as long as 6 tones (600 ms) in this manner and that the detection of the emergence of a regular pattern is remarkably efficient (see also Deutsch, 1975; Warren and Byrnes, 1975; Agus et al., 2010).

#### The timing of change perception

Given the RT figures measured in the DETECT block, we sought to determine when listeners *perceive* the onset of a RAND-REG transition: Do they perceive the onset of the RAND-REG transition at the point when they detected it (879 ms) or do they re-adjust the perceived time of change closer to the nominal transition? Using a light flash as an external time marker we instructed subjects to determine the temporal relationship of the flash relative to the stimulus transition. This procedure enabled us to determine listeners’ PSS – the abscissa at 0.5 of the psychometric fit. That is, the point at which they are maximally uncertain about the temporal order and report “flash before” and “flash after” equally frequently (operationally this is thus defined as the point at which they perceive the light flash as simultaneous with the stimulus change).

In this task, REG-RAND signals serve as controls for the RAND-REG stimuli. Since the REG-RAND transition is clearly temporally defined, the PSS for REG-RAND stimuli should be aligned with the nominal transition. A PSS value that is different from 0 would be attributable to the bias (Stone et al., 2001; van Eijk et al., 2008) or other error involved in comparing the timing of audio and visual events and the PSS for RAND-REG stimuli can then be adjusted accordingly.

Figure 2 presents the average performance across subjects. The *x*-axis represents the time of the flash relative to the nominal transition (negative numbers indicate flash before transition) and the *y*-axis represents the proportion of trials for which the flash was judged to occur later than the acoustic transition. The black curves in Figures 2A,B are cumulative Gaussian fits to the mean data for the REG-RAND and RAND-REG transitions respectively. The gray lines are fits obtained in each of 1000 bootstrap iterations. The spread of bootstrap fits indicates the variability of the data. Figure 2C plots REG-RAND and RAND-REG fits on the same plot to demonstrate that the slopes are very similar, suggesting that subjects transitioned between a “*flash before*” and “flash *after*” percept at an equal speed for both stimulus types, further confirming that REG-RAND is a useful control. The mean PSS in the REG-RAND condition is 76 ms (SD = 50 ms), suggesting that subjects perceived that transition as simultaneous with the light flash if that flash followed it by 0.7 sound pips. The direction of the bias is different from the negative bias usually reported in the audio-visual simultaneity literature (but note that most previous studies employed much simpler signals; Stone et al., 2001; King, 2005; van Eijk et al., 2008). It is also different from the bias we measured with a very similar setup previously (Patel and Chait, 2011) and likely reflects large inter-subject variability which usually characterizes audio-visual simultaneity measurements (Stone et al., 2001). Importantly, however, the difference between REG-RAND and RAND-REG is consistent across subjects (see below).

The mean PSS in the RAND-REG condition was 460 ms (SD = 96 ms) implying that subjects perceive the transition and light flash as simultaneous when the light *succeeds* the RAND-REG transition by 4.6 sound pips. Adjusting this value by the PSS value obtained for the REG-RAND stimuli results in a corrected value of: 460 − 76 = 384 ms, which is significantly shorter than the RAND-REG detection time of 879 ms obtained from the data in the DETECT block (marked with an arrow in Figure 2C).

To evaluate the degree to which the corrected PSS value is consistent across subjects, Figure 3A shows a histogram computed with a repeated measures bootstrap routine. Similarly to the average-fit-based PSS, the histogram peaks at 380 ms, with a rather narrow standard deviation (75 ms). In Figure 3B we evaluate the distribution of temporal readjustment by subtracting, for each subject, their adjusted PSS from the detection time (estimated based on RT difference between REG-RAND and RAND-REG, as described above). This reveals a, fairly narrowly distributed, mean recalibration size of 470 ms.

The data thus suggest that listeners do not perceive the transition in the RAND-REG stimuli to have occurred at the point of detection, but rather re-adjust the perceived time of occurrence, by shifting it about 470 ms “back in time” toward the nominal transition. These results replicate data we have reported previously (Patel and Chait, 2011) for tone sequences with a regularity cycle of 4 tones. It is notable that this readjustment is not complete, but rather listeners only readjust the percept to about half way between detection time and nominal change time. Since the distribution of readjustment is rather narrow (Figure 3B) it is likely that the limitation stems from fairly low level mechanisms. One possibility is that this reflects limits on some form of fixed capacity working memory store used for the task. To investigate the properties of this putative memory store, and specifically whether it might be limited by absolute time or by stimulus characteristics (number of tone pips), in Experiment 2 we shortened the tone pips by 50%.

## Experiment 2

In Experiment 2, we investigate the temporal capacity of the temporal readjustment process by shortening the pip duration from 100 to 50 ms, which would halve the detection time. If the observed effects are based on a fixed-duration echoic memory store, the readjustment should be nearly complete. Else, if the memory store is limited by number of events, rather than absolute duration, we expect to observe effects that are qualitatively similar to those in Experiment 1, but scaled by half.

### Methods

#### Participants

Ten paid subjects (mean age 21.1, five males) participated in the experiment. Three of the subjects also participated in Experiment 1. All participants reported normal hearing, and no history of neurological disorders. The experimental procedures were approved by the UCL ethics committee and written informed consent was obtained from each participant.

#### Stimuli

The stimuli were as those in Experiment 1 except that pip duration was shortened to 50 ms. The light flashes were timed to coincide with individual pips and therefore ranged in latency from −500 ms (10 pips before the transition) to +700 ms (14 pips after the transition).

### Results

#### The timing of change detection

In the DETECT block, the average RT measured for REG-RAND transitions was 613 ms (SD = 87). While this appears to be lower than the mean RT measured for the REG-RAND condition in Experiment 1, the difference is not statistically significant (independent sample *t* test, *p* = 0.175), as expected since the detection of the REG-RAND transition should not depend on changing pip duration. The mean RT for RAND-REG was 1115 ms (SD = 60), and the RT difference, which will provide a lower bound measure of the computation time required to detect the emergence of regularity in RAND-REG, is therefore 502 ms (a duration of 10 tone pips). Here again the data demonstrate that listeners require about a cycle and a half (6 + 4 tones) to detect the emergence of a repeating pattern.

#### The timing of change perception

Figure 4 presents the average performance in Experiment 2. The plots are narrower than those in Figure 2, because the flashes are timed to coincide with pips and thus occurred closer, in absolute time, to the transition. The *x*-axis represents the time of the flash relative to the nominal transition (negative numbers indicate flash before transition) and the *y*-axis represents the proportion of trials for which the flash was judged to occur later than the acoustic transition. The mean PSS in the REG-RAND condition is −39 ms (SD = 41 ms). The mean PSS in the RAND-REG condition was 193 ms (SD = 55 ms) implying that subjects perceive the transition and light flash as simultaneous when the light *succeeds* the RAND-REG transition by 3.8 tone pips. Adjusting this value by the PSS value obtained for the REG-RAND stimuli results in a corrected value of: 193 − (−39) = 232 ms, which is significantly shorter than the RAND-REG detection time of 502 ms obtained from the data in the DETECT block (marked with an arrow in Figure 4C).

Similarly to Figure 3 in Experiment 1, Figure 5 shows a bootstrap-based histogram of PSS values. The histogram peaks at 230 ms, with a rather narrow standard deviation (55 ms). In Figure 5B we evaluate the distribution of temporal readjustment. This similarly indicates a fairly narrowly distributed, mean recalibration size of 285 ms. Namely, listeners readjusted the perceived time by moving it 5.7 tones back toward the nominal transition.

Were the recalibration based on some form of fixed capacity memory store, the results of Experiment 1 would suggest that the size of this store is about 470 ms (the size of the recalibration in that experiment) and we would therefore expect the recalibration to be essentially perfect in Experiment 2 (where the distance between the nominal transition and the detection time is about 500 ms). However, the results demonstrate that in Experiment 2 the recalibration is similarly incomplete. In effect, the magnitude of the recalibration, when measured in number of pips, is comparable in both experiments, suggesting that temporal readjustment depends on this property of the signal.

### Discussion

The TOJ task used here required listeners to register the times of occurrence of the flash and transition and then determine their relative order. To make sure that the decision is based on knowledge of both event times (rather than only the event that happened first), listeners were instructed to respond following the end of the auditory stimulus, only after both events had occurred. When subjects were interviewed, at the end of the experiment, they did not report noticing timing mismatches or actively time-shifting their estimates. It is critical to note that the stimuli are far too rapid for any conscious scanning and evidence accumulation. Namely, listeners do not detect the change by explicitly comparing incoming tones to a remembered sequence. The transition (in REG-RAND or RAND-REG) pops out automatically irrespective of any deliberate effort (see Demo Sounds in Supplementary Material). Similarly, due to the fast tone-succession rate, it is impossible to solve the time-estimation task by explicitly (consciously) “backward scanning” the sequence. Rather the task involves comparing the timing of two bottom-up generated sensory events. Indeed, Figures 2C and 4C demonstrate that, save for the temporal displacement, the psychometric curve for the RAND-REG transition is very similar to that of REG-RAND, suggesting that listeners used a similar criterion for the order judgment in the two stimuli (that is to say, they were equally confident about the order in both cases). This is a key point because the REG-RAND transition is precisely defined in time (as the first tone pip that violates the preceding regularity pattern) and the similarity thus suggests that the temporal order in RAND-REG was as perceptually clear as for REG-RAND. The most likely explanation of the data therefore points to pre-attentive readjustment of perceived timing.

Our results, together with those from Patel and Chait (2011), demonstrate a phenomenon that is likely to play a major role in an organism’s struggle to make sense of dynamic environments. We show that listeners do not perceive the time of occurrence of RAND-REG events at the point when they were detected, but instead consistently re-adjust the perceived time backward toward the nominal transition time. The size of this recalibration appears to not be determined by a fixed-duration memory store but rather on number of events (i.e., tone pips) in the regular pattern. However, while readjustment reliably takes place, listeners only readjust to about halfway (just under one cycle) between detection time and the nominal transition. The incompleteness of the readjustment cannot be due to limits on auditory memory – performance on the DETECT task in the present experiment, which is also dependent on memory of the on-going sequence, demonstrated that the system is able to store a sequence with a duration of at least 600 ms (6 tone pips). In Patel and Chait (2011) the stimuli comprised of 100 ms tones with regularity cycles of 4 tones. Detection time for RAND-REG was estimated to be 537 ms (6 tones). If recalibration were only limited by auditory memory capacity, we would have expected an essentially complete readjustment in that experiment, contrary to what was actually observed. It is also unlikely that the incomplete adjustment is caused by depleted visual memory (difficulty remembering the timing of the flash) since this explanation would predict better performance in Experiment 2 (where the absolute time between detection and transition is shorter) – an effect which is not observed in the present data. It may be that performance is limited by some form of auditory-visual memory required for the task – e.g., Inaccurate ability to identify the relative time of the visual flash and transition (which auditory tone the flash coincided with).Alternatively partial readjustment (only about one cycle backward) could reflect a genuine mechanism for preventing situations of over-readjustment. Indeed, partial readjustment may be sufficient for natural scenes where the difference between detection time and nominal change time are significantly shorter than in the signals used here.

The stimuli used in the present experiment are obviously highly un-natural, with a very long regularity cycle that is unlikely to occur in natural scenes. We use these long patterns in order to reliably distinguish the perceptual detection time (measured via RT) from the nominal transition. Only when these are discernible and spaced widely enough apart, can any potential recalibration be measured. Regular patterns in natural sound sequences are likely characterized by periods that are at least an order of magnitude shorter (this is an estimate, as there are currently no data specifically measuring these kinds of statistics), requiring much smaller readjustment. It is therefore noteworthy that, in our experiments listeners demonstrated the ability to recalibrate over time spans much longer than those encountered in the everyday environment.

## Conflict of Interest Statement

The authors declare that the research was conducted in the absence of any commercial or financial relationships that could be construed as a potential conflict of interest.

## Supplementary Material

The Supplementary Material for this article can be found online at http://www.frontiersin.org/Auditory_Cognitive_Neuroscience/10.3389/fpsyg.2012.00396/abstract

## Footnotes

^1^http://www.vislab.ucl.ac.uk/cogent.php

^2^http://bootstrap-software.org/psignifit/
